# Prevalence and biopsychosocial factors associated with depressive symptoms among patients living with systemic lupus erythematosus in clinical settings in urban Thailand

**DOI:** 10.1186/s12888-022-03739-z

**Published:** 2022-02-09

**Authors:** Nirunya Narupan, Acharaporn Seeherunwong, Walailak Pumpuang

**Affiliations:** 1 M.N.S. Candidate, Faculty of Nursing, Mahidol University, Bangkok, Thailand; 2 Department of Mental Health and Psychiatric Nursing, Faculty of Nursing, Mahidol University, Bangkok, Thailand

**Keywords:** Systemic lupus erythematosus, Disease flare, Organ damage, Pain, Fatigue, Steroid dosage, Body image, Social support, Depressive symptoms

## Abstract

**Background:**

Depressive symptoms are globally recognized as a significant mental health problem in patients with chronic disease, particularly those with systemic lupus erythematosus (SLE). The purpose of this study was to estimate the prevalence and examine biopsychosocial factors of depressive symptoms among patients with SLE.

**Methods:**

This cross-sectional study was conducted among 185 participants diagnosed with SLE and received treatment for at least 3 months, aged 18–59 years attending the outpatient clinic of a university hospital, Bangkok, Thailand. Depressive symptoms were measured by the Thai version of the Patient Health Questionnaire-9. We assessed Demographic data, the Systemic Lupus Erythematosus Activity Index, the Systemic Lupus International Collaborating Clinics Damage Index, Numeric Rating Scale, Fatigue Severity Scale, Body Image Scale, and the ENRICHD Social Support Instrument. Data were collected from March to May 2021. Multivariable logistic regression was used to analyze the data.

**Results:**

The proportion of the participants with depressive symptoms was 43.2%, which 8.1% of those patients presented moderate to severe depressive symptoms. In a multivariable logistic regression model, SLE patients with depressive symptoms were more likely to be severe pain (aOR = 12.11, 95% CI: 1.35, 108.46), fatigue (aOR = 2.36, 95%CI: 1.08, 5.14), taking prednisolone ≥15 mg daily (aOR = 5.75, 95%CI: 1.76, 18.80), low satisfied of body image (aOR = 12.49, 95%CI: 2.23, 69.80), and low social support (aOR = 17.96, 95% CI: 1.86, 173.77). Disease flare, organ damage, and family income sufficiency did not significantly increase the risk of depressive symptoms in patients with SLE.

**Conclusions:**

The findings highlight depressive symptoms in patients with SLE. Therefore, the health professional should be concerned about the perception of body image, level of social support, fatigue, and pain while treating patients with SLE. Public health screening programs to identify depressive symptoms in patients with SLE are needed. In addition, a high dose of prednisolone should be considered if required, along with monitoring.

## Introduction

Depression is the most frequent among neuropsychiatric manifestation complaints in patients with Systemic Lupus Erythematosus (SLE) [1, 2] that impairs daily living for sufferers and causes significant societal and economic burden [3, 4]. The high incidence and increasing prevalence of depression in patients with SLE has been recognized as a severe mental health problem of non-communicable diseases (NCDs) of the twenty-first century [5, 6]. Point prevalence rates range from 2 to 91.7%, depending upon the context, setting, and assessment tool [2, 7]. Eight to 24% of patients were diagnosed with depressive disorders [7, 8], and 12% had suicidal thoughts [9]. A previous study in northern Thailand revealed that the prevalence of depressive symptoms among patients with SLE was 45.2% [10]. Evidence suggests many factors are significant predictors of depression in patients with SLE [2, 11]. Therefore, including multiple factors into Engel’s biopsychosocial model of depression should create a fuller picture of the pathophysiology of depressive symptoms in patients with SLE [12].

SLE is the prototypical autoimmune disease affecting multi-organ systems. A complex interaction of genetics, environment, and hormones leads to immune dysregulation and breakdown of tolerance to self-antigens, resulting in autoantibody production, inflammation, and destruction of end-organs. It is a significant disease burden across the world among different ethnic, racial, and age groups [13]. SLE troubles up to 12 people per 5000 worldwide [14], and its incidence is 0.9 to 3.1 cases per 100,000 population per year [15]. Usually, patients with SLE are diagnosed in early adulthood. Cases of this disease are 80 to 90% female between the ages of 20 and 40 years (mean age at diagnosis: 29 years) [16]. They are more common in Afro-Caribbean, Chinese, and Asian populations than Caucasians [17].

The nature of SLE is a complex disease that can affect the body without limitation, and the disease causes many different clinical symptoms [16]. Typically, patients with SLE have an inflammatory illness that occurs in various organ systems. In patients with SLE, involvement of the central nervous system (CNS) is associated with a worse prognosis and more cumulative damage as neuropsychiatric systemic lupus erythematosus (NPSLE), with psychiatric disease manifestations [18], which are more likely to elevate serum levels of antibodies [19] and demonstrate a significant association with several genotypical pathways [20]. NPSLE is identified by the American College of Rheumatology (ACR) [21]. At the same time, patients with this disease need treatment with long-term monitoring of symptoms since the diagnosis of SLE is more accurate nowadays. SLE treatment requires adjusting the level of drug therapy per the results of biomarkers and disease manifestations and impacts on SLE that signal appropriate treatment adjustments. However, one treatment may not continuously address the patient’s overall health condition due to the generalized and chronic nature of the disease. This state has long-term effects, with significant impacts on physical and mental health, including the patient’s quality of life [16, 22]. A previous study reported that more than 2 of 3 patients with SLE experienced emotional illness such as sadness, depression, fear, anxiety, guilt, anger, and wrath [16]. These morbidities seriously affect behaviors and may lead to significant psychological problems [9].

**Fig. 1:**
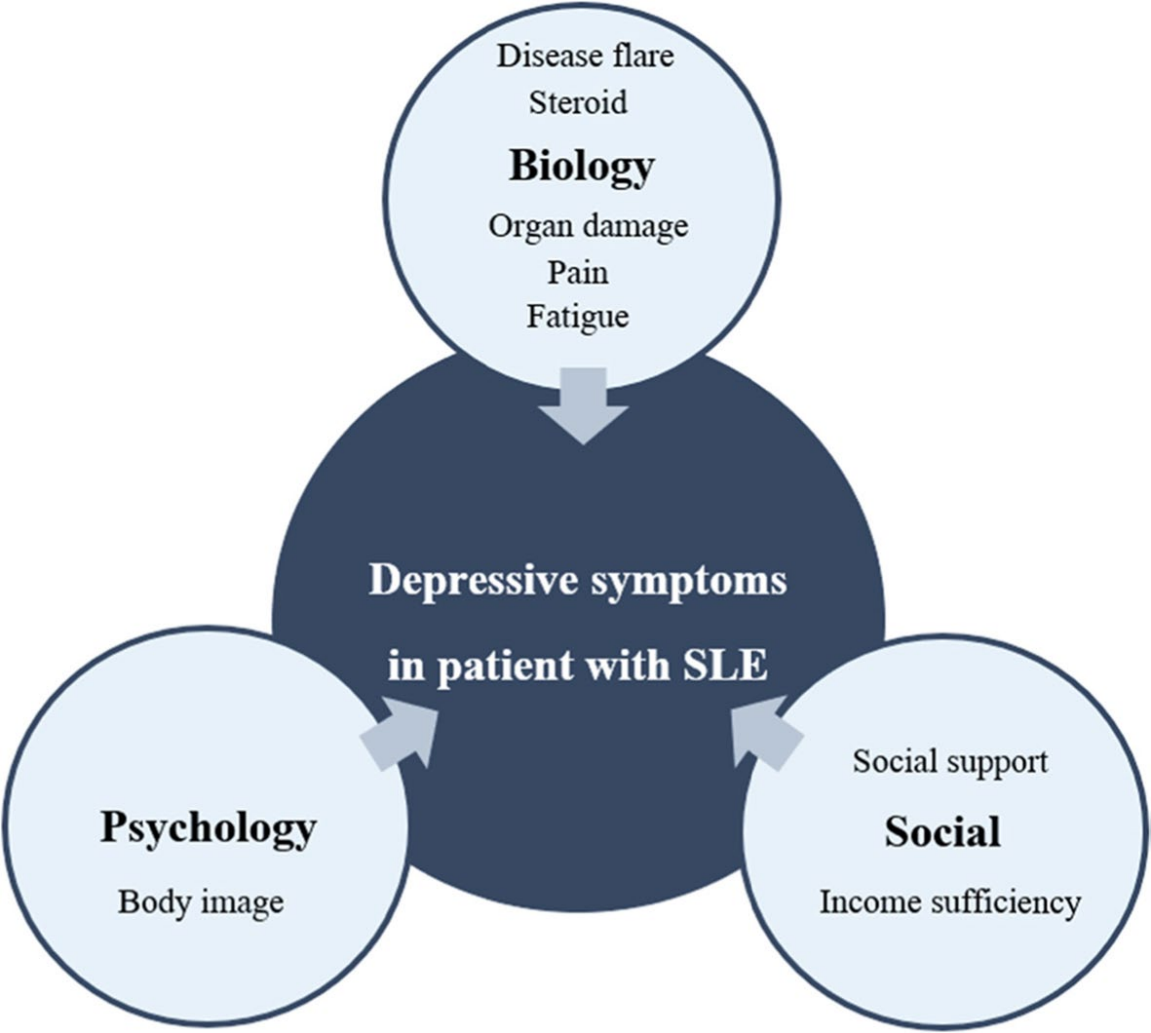
Biopsychosocial factors of depressive symptoms in patients with SLE

Depression is a profoundly impactful comorbidity for SLE patients’ health and well-being [23], with common manifestations such as higher levels of fatigue, more significant pain, and poor sleep quality [24–26]. Pathogenesis of depressive symptoms in SLE is also expected and involves complex interactions between cytokines, antibodies, the role of genes, etc. In the same way, the result of damage accrual, cumulative glucocorticoid use, psychology status, and social-supportive condition are essentially triggered [2, 11]. Biopsychosocial factors that are critical and related to the depressive symptoms of patients with SLE are presented in Fig. 1.

Malfunction and injuries in various body organs are essential factors that cause patients to face grave disease prognoses. The severity of SLE, including active disease or disease flare and disability from organ damage [27, 28], and pain and fatigue increase the risk of depressive symptoms [29]. Changes in physiology and the immune system are common in patients with SLE. Patients with SLE fear the disease progression, another aggravation or spreading to new organs, and insecurity regarding their future life. These fears are often related to unpleasant experiences [16, 30]. Fatigue and pain are common symptoms in SLE; 50 to 90% of patients with SLE experience constant fatigue [16], and over 90% of patients with SLE suffer from joint pain [16, 31]. These conditions cause patients to struggle with fatigue as paralyzing, insurmountable with sleep or rest. They limit everyday activities, often forcing patients to resign from earlier interests, hobbies, or work. Sometimes, patients feel helpless, powerless, angry, and guilty. Therefore, fatigue and pain were the two most reported symptoms affecting patients’ quality of life with SLE, limiting everyday life activities [32]. According to past findings, fatigue was a significant contributor to depression in patients with SLE [29], and over 82% of patients with depressive symptoms experienced moderate to severe pain [33].

Patients with SLE are often treated with glucocorticoids as monotherapy or combination therapy with hydroxychloroquine, non-steroidal anti-inflammatory drugs (NSAIDs), and immunosuppressants. Studies show that immunosuppressants (methotrexate, cyclophosphamide) reduce disease activity [34]. However, the unpleasant side effects of high doses of steroid therapy can cause symptoms of neuropsychiatric lupus but more often result in milder emotional changes, such as anxiety or depression. For example, continually taking more than 15 to 40 mg of prednisolone per day predicted depression in patients with SLE [22, 27, 35].

Changing physical appearance from disease progression and its treatment leads to low self-esteem and increases the risk of depressive symptoms [36, 37]. Unsightly skin lesions such as classic erythema on the face, discoid rash, lesions with a tendency to scarring, skin atrophy, and hair loss are symptoms that cause patients with SLE to feel embarrassed. Patients also report bruising susceptibility and increased photosensitivity. In addition, the side effect of glucocorticosteroid medicine also is a cause of unsightly skin lesions and obesity [31]. Appearance changes may make patients feel less attractive and cause concern about adverse reactions from their partners [31]. As a result, many patients tend to lose confidence and have lower satisfaction with their self-image.

Living with SLE hurts partnerships, family relationships, and social life. Due to increasing disability, patients spend less overall and quality time with their partners [33]. The unpredictable course of SLE is why patients’ social life is limited, facing rejection and increasing isolation. Patients with SLE who lack good social support often face conflicts with family members that cause mental health problems, particularly depressive symptoms [28]. In contrast, relevant support ensured by the family or close persons helps patients avoid excessive burden [22].

Poor financial status or insufficiency of household income is a factor associated with depressive symptoms in patients with SLE [38]. After 5 years with SLE, 15 to 40% of patients had lost their jobs, and after 10 or 15 years, 36 and 52% were jobless [16, 39]. Many patients with SLE are distressed that the disease will adversely affect their planned development path [16]. The patients are also concerned about illness costs, such as medical and additional healthcare insurance costs [32].

Depressive symptoms in patients with SLE have been examined in previous studies; however, findings were with Caucasian patients [2] that may exhibit characteristics different than in Asian patients, including Thais [40]. Asians with SLE have more severe clinical manifestations than Caucasian patients [41–43]. In meta-analysis studies on various gene polymorphisms, the FcgRIIIA-F158 allele is associated with low binding affinity to IgG1 and IgG3 in Asian patients [43]. Asian patients with SLE also have higher rates of renal involvement-associated autoantibodies when compared with Caucasians, and that often exhibit a more severe disease flare [43]. Access to health care is a crucial determinant of disease progression, treatment outcome, and the management of complications, particularly in Asian patients [43]. Thus, the difference between Asians with SLE and other ethnicities may influence disease development. In addition, most studies focused on analyzing only the psychological factors [2]. The analysis of biopsychosocial factors simultaneously was rare. These kinds of results are not sufficiently conclusive for clinical application.

The main objective of the present study was to determine biopsychosocial factors associated with depressive symptoms among patients living with SLE in clinical settings in urban Thailand. In addition, the prevalence of depressive symptoms was estimated.

## Methods

A cross-sectional study was conducted in three outpatient medical clinics, including the medicine, nephrology, and rheumatology clinics of a university hospital in Bangkok, Thailand.

### Setting

Thailand is an upper-middle-income country located in South-East Asia. The current population of Thailand is 70,005,912 based on Worldometer elaboration of the latest United Nations data [44]. 51.1% of the population is urban, adult, with a median age of 40.1 years [44]. The National Statistical Office of Thailand (NSO) reported that more than 1 in 5 of Thailand’s population health has some chronic illness or congenital disease [45]. Most of the Thai population has government health care benefits. Most use universal health insurance (gold card), followed by social security, civil servant, or state enterprise health benefits. We conducted this study at a university hospital established in 1914 in Bangkok city, the capital of Thailand. The hospital is a tertiary hospital governed by the Thai Red Cross Society and affiliated with the faculty of medicine with inpatient services of 1435 beds. This hospital is a training institute in physical health and mental health for expertise by health professionals and readiness of medical devices for complex illness. The total number of patients with SLE (ICD-10: M32) reported by information technology data management of the hospital in 2019–2020 was more than 1098 SLE patients admitted to the outpatient department. They get a physical exam, standard treatment, and assessment by the division of rheumatology from specialists. Patients with SLE enrolled in this study were followed through the outpatient department in three clinics, internal medicine, kidney, and rheumatology clinics.

Data collection operations took place during the COVID-19 outbreak. The researcher and participants strictly complied with the rules for preventing the spread of infection by completing questionnaires and body temperature checks, always wearing masks, and keeping social distancing of at least 2 m.

### Participants

Patients with SLE were recruited at the time of their routine follow-up visit. The patients were eligible if between 18 and 59 years old, both male and female, and diagnosed according to the 1997 ACR, Revised Criteria for the classification of Systemic Lupus Erythematosus and were on medication for at least 3 months, patients observed with stable treatment. All patients were Thai, and able to read and write and give informed consent in the Thai language. Those who were unable to provide essential knowledge required in the study protocol, lacked necessary communication skills, were diagnosed with psychiatric disorders, had comorbid physical illnesses of life-threatening conditions, or need urgent treatment were excluded from the study. Inclusion and exclusion criteria were affirmed for essential information by interview and medical record.

The sample size was calculated by G*Power version 3.1.9.7 program and based on the results of a study, an independent pain variable by Chang et al. [38] depression and quality of life in patients with systemic lupus erythematosus: odd = 3.477, two tails x distribution equal to the binomial, confidence level in test α = 0.05, power of test = 0.80, the relationship between other variables (R2 other X) = 0, and X parm π = 0.638, at least 168 participants were needed. Adding 10.0% to account for error during the study, at least 185 participants were required for the analysis.

### Instruments

The socio-demographic and medical history questionnaire was developed by researchers and reviewed by a panel of three experts and yielded a content validity score of 1, which consisted of 19 items divided into two parts:

- Part 1: Personal information contains items about sex, age, religion, marital status, education level, occupation, rights to medical treatment, family characteristics, number of family members, average family income per month, and the sufficiency of household income.
- Part 2: Clinical information contains items about the duration of SLE disease, daily steroid dosage, disease flare, other congenital diseases, history of mental illness, weight, height, and body mass index.

### Assessment of depressive symptoms

Depressive symptoms were evaluated with the Thai version of the nine-item Patient Health Questionnaire (PHQ-9) [46]. The Thai version of the PHQ-9 was translated from the original PHQ-9 [47]. The PHQ-9 is a self-report measure consisting of 9 questions based on the 9 DSM-IV criteria for major depressive episodes. The respondents rated the symptoms experienced during the prior 2 weeks. Scores for each item range from 0 (not at all) to 1 (several days), 2 (more than half of the days), and 3 (nearly every day), while summed scores range from 0 to 27. The total scores were classified as indicating the level of severity of depressive symptoms: mild (5 to 9), moderate (10 to 14), moderately severe (15 to 19), and severe depression (20 and over). The Thai version of the PHQ-9 has acceptable psychometric properties for screening for major depression in general practice, with a recommended cut-off score of nine or more. Cronbach’s alpha for the total scale was 0.79, and predictive validity was 0.96 [46].

### Measures of biological predictors

The Systemic Lupus Erythematosus Activity Index (SLEDAI), developed by Bombardier et al. [48], was used to assess disease fare. This instrument collected data from participants’ medical records by the researcher trained by a nephrologist. The SLEDAI consists of 24 questions about symptoms and laboratory results. The answer characteristic is a rating scale ranging from 1, 2, 4, and 8 points. In this study, disease flare was divided into two groups: the non-disease flare group, which scored less than 3 points, and the disease flare group, three or higher.

The Systemic Lupus International Collaborating Clinics Damage Index (SLICC Damage Index) developed by Gladman et al. [49] was used to assess organ damage. The SLICC damage index consists of 12 questions about damage to 12 organ systems in the body, and the answer characteristic is a rating scale ranging from 1 to 2 points. In this study, organ damage was divided into two groups: the non-organ damaged group: 0 points, and the group with organ damage: the score was 1 point or higher. This instrument collected data from participants’ medical records by the researcher trained by a nephrologist.

The Numeric Rating Scale (NRS) was used to assess the level of pain, with a scale developed by Jensen & Karoly [50] and updated by the Thai association for the study of pain (2009). It had 1 question each on position and pain level using a numeric scale from 0 to 10. In this study, the pain was divided into four groups: the group with no pain: the score was 0 points, the group with mild pain: the score was 1 to 3 points, the group with moderate pain: the score was 4 to 6 points, and the group with severe pain: the score was 7 points or higher. The pain assessment was adjusted by self-report of participants.

Fatigue Severity Scale (FSS) developed by Krupp et al. [51] and translated into a Thai version by Sawasdee [52] measured fatigue severity. The FSS consists of 9 questions, eight levels of Likert scale from 1 to 7 points. The score is calculated based on the answers divided by the total number of questions. In this study, fatigue was split into two groups: the group without fatigue: the score was less than 4 points, and the fatigue group was 4 points or higher. Content validity by a panel of three experts yielded a CVI score of 1. The internal consistency in those living with SLE (*n* = 30) obtained a Cronbach’s alpha coefficient of .89. The fatigue assessment was adjusted by self-report of participants.

### Measures of psychological predictor

Body Image Scale (BIS) measured the level of body-image satisfaction as developed by Hopwood et al. [53] and was translated into the Thai version by Cheewapoonpol [54]. The BIS consists of 10 negative questions about satisfaction with one’s physical image, and the answer is a rating scale of 4 levels from 1 to 4 points. In this study, there were three groups of physical image satisfaction groups: the group with low level: the score was 31 to 40 points, the group with moderate level: the score was 21 to 30 points, and the group with high level: the score was 10 to 20 points. A panel of three experts rated content validity yielded a CVI score of 1. The internal consistency in those living with SLE (*n* = 30) showed a Cronbach’s alpha coefficient of .89. The body image assessment was a self-report of participants.

### The social predictor measure

The ENRICHD Social Support Instrument (ESSI) measured social support as developed by Mitchell et al. [55] and translated into the Thai version by Lortajakul [56]. The ESSI consists of 7 questions, and the answer is a rating scale of 5 levels from 1 to 5 points. In this study, there were two groups of social support: the group with mild social support: the score was 1 to 10 points, the group with moderate social support: the score was 11 to 20 points, and the group with high social support: the score was 21 to 30 points. Content validity by a panel of three experts yielded a CVI score of .95. The internal consistency in those living with SLE (*n* = 30) obtained a Cronbach’s alpha coefficient of .94. The social support assessment was adjusted by self-report of participants.

### Ethical consideration

Ethical approval was granted by the Institutional Review Board Faculty of Nursing, Mahidol University (COA No. IRB-NS2020/583.1812), and the Institutional Review Board Faculty of Medicine, Chulalongkorn University, Thailand (COA No. 310/2021). In addition, the committee for research of the hospital approved the research project before working with human subjects. Participants completed informed consent forms. The researcher also asked subjects’ permission to use the data contained in their medical records. All procedures were performed following ethical guidelines and regulations. The study was conducted from November 2020 through July 2021, and participant recruitment took place from March 2021 to May 2021. The participants answered six questionnaires by themselves, including 48 questions, the data collection produced approximately 20 to 25 min for each participant.

### Data analysis

Data were double entered into an Excel sheet before being transferred into the SPSS program version 22.0 for analysis. Categorical and continuous data were appropriately analyzed to present the characteristics of participants by descriptive statistics. The chi-square test assessed characteristics between patients with and without depressive symptoms. Univariate logistic regression analysis was performed per each biopsychosocial variable. The complete multivariable logistic regression analysis model included all significant independent variables at a significance level of α = 0.05.

## Results

### Personal characteristics of the participants

The study comprised 185 Thai men and women who were diagnosed with SLE. Most of them were female (96.2%), between 36 and 59 years old (70.3%), with an average age of 42.16 years (SD = 10.78), single (52.4%), Buddhists (95.7%), and had bachelor’s degrees (42.7%). Most of the patients work (72%) and use the social security schemes to access healthcare (38.9%). Most of the patients are dwelling in single families (81.1%) with 1 to 3 of family members (57.8%), the average household monthly incomes were between 10,000 and 25,000 baht (34.1%) and had no income leftover (51.4%) (Table 1).

**Table 1:** Personal characteristics of the participants with or without depressive symptoms (*n* = 185)

	Total (%)	Depressed	Non-depressed	*p*-value
		N (%)	N (%)	
**Sex**				
Female	178 (96.2)	79 (44.4)	99 (55.6)	0.142‡
Male	7 (3.8)	1 (14.3)	6 (85.7)	
**Age (year)**				
18–35	55 (29.7)	24 (43.6)	31 (56.4)	0.944^†^
36–59	130 (70.3)	56 (43.1)	74 (56.9)	
(Min = 19, Max = 59, Mean = 42.16, SD = 10.78)				
**Marital status**				
Single	97 (52.4)	46 (47.4)	51 (52.6)	0.051‡
Married	69 (37.3)	22 (31.9)	47 (68.1)	
Divorce	12 (6.5)	8 (66.7)	4 (33.3)	
Widowed	7 (3.8)	4 (57.1)	3 (42.9)	
**Religion**				
Buddhism	177 (95.7)	75 (42.4)	102 (57.6)	0.157‡
Christianity	4 (2.2)	2 (50.0)	2 (50.0)	
Islam	3 (1.6)	3 (100.0)	0 (0)	
Hindu	1 (.5)	0 (0)	1 (100.0)	
**Education**				
No formal education	5 (2.7)	3 (60.0)	2 (40.0)	0.553^†^
Primary	20 (10.8)	11 (55.0)	9 (45.0)	
Secondary	65 (35.1)	30 (46.2)	35 (53.8)	
Bachelor	79 (42.7)	30 (38.0)	49 (62.0)	
Post-graduate	16 (8.6)	6 (37.5)	10 (62.5)	
**Occupation**				
Unemployment	52 (28.1)	27 (51.9)	25 (48.1)	0.645^†^
Civil servant	36 (19.5)	13 (36.1)	23 (63.9)	
Employee	35 (18.9)	15 (42.9)	20 (57.1)	
Self-employed	34 (18.4)	12 (35.3)	22 (64.7)	
Labor	24 (13.0)	11 (45.8)	13 (54.2)	
Agriculturist	4 (2.2)	2 (50.0)	2 (50.0)	
**Right to access healthcare**				
Social security scheme	72 (38.9)	28 (38.9)	44 (61.1)	0.509^†^
Universal scheme	68 (36.8)	33 (48.5)	35 (51.5)	
Cash or insurance	45 (24.3)	19 (42.2)	26 (57.8)	
**Family**				
Single	150 (81.1)	66 (44.0)	84 (56.0)	0.667‡
Extend	35 (18.9)	14 (40.0)	21 (60.0)	
**Family members (persons)**				
1–3	107 (57.8)	51 (47.7)	56 (52.3)	0.166^†^
4–5	60 (32.4)	20 (33.3)	40 (66.7)	
> 5	18 (9.7)	9 (50.0)	9 (50.0)	
(Min = 1, Max = 11, Mean = 3.54, SD = 1.72)				
**Household monthly income (baht)**				
< 10,000	5 (2.7)	4 (80.0)	1 (20.0)	0.262^†^
10,000–25,000	63 (34.1)	31 (49.2)	32 (50.8)	
25,001–40,000	38 (20.5)	16 (42.1)	22 (57.9)	
40,001–60,000	46 (24.9)	17 (37.0)	29 (63.0)	
> 60,000	33 (17.8)	12 (36.4)	21 (63.6)	
(Min = 3000, Max = 200,000, Mean = 45,262.76, SD = 35,516.22)				
**Household income sufficiency**				
Leftover	90 (48.6)	30 (33.3)	60 (66.7)	0.008^†^
No leftover	95 (51.4)	50 (52.6)	45 (47.4)	

^†^the *p*-value associated with Chi-square test

^‡^*p*-value from Fisher’s Exact test

### Clinical characteristics of the participants

Most of the patients had been diagnosed with SLE for 11 to 20 years (36.8%), took prednisolone 1 to 5 mg daily (48.1%), and had disease remission (78.4%). The result showed that 161 (87% of patients) had other congenital diseases, for instance, lupus nephritis (47%), hypertension (19.5%), and dyslipidemia (13.5%). They had an average body image index of 23.10 kg/m^2^ (SD =5.77), 94 (50.9%) of patients were of abnormal weight, with 16.8% underweight and 34.1% overweight (Table 2).

**Table 2:** Clinical characteristics of the participants with or without depressive symptoms (*n* = 185)

	Total	Depressed	Non-depressed	*p*-value
		N (%)	N (%)	
**Duration of disease (year)**				
≤ 5	39 (21.1)	17 (43.6)	22 (56.4)	0.847^†^
6–10	51 (27.6)	20 (39.2)	31 (60.8)	
11–20	68 (36.8)	32 (47.1)	36 (52.9)	
> 20	27 (14.6)	11 (40.7)	16 (59.3)	
(Min = 0.50, Max = 38, Mean = 12.84, SD = 8.09)				
**Prednisolone (mg/day)**				
Unused	51 (27.6)	18 (35.3)	33 (64.7)	0.004^†^
1–5	89 (48.1)	32 (36.0)	57 (64.0)	
6–14	13 (7.0)	8 (61.5)	5 (38.5)	
≥ 15	32 (17.3)	22 (68.8)	10 (31.2)	
(Min = 0, Max = 40, Mean = 5.90, SD = 7.71)				
**Disease flares**				
Non-disease flare	145 (78.4)	54 (37.2)	91 (62.8)	0.005^†^
Disease flare	40 (21.6)	26 (65.0)	14 (35.0)	
**Disease flares in body systems (≥ 1 answer)**				
Kidney system	16 (8.6)	12 (75.0)	4 (25.0)	0.007^†^
Muscle and joints	7 (3.8)	6 (85.7)	1 (14.3)	0.044‡
Hematology	7 (3.8)	5 (71.4)	2 (28.6)	0.242‡
Skin	11 (5.9)	5 (45.5)	6 (54.5)	1.000‡
**Other congenital disease**s				
Nom-congenital disease	24 (13.0)	8 (33.3)	16 (66.7)	0.294^†^
Have congenital disease	161 (87.0)	72 (44.7)	89 (55.3)	
**Congenital disease (**≥ **1 answer)**				
Lupus nephritis	87 (47.0)	36 (37.6)	51 (49.4)	0.630^†^
Hypertension	36 (19.5)	16 (44.4)	20 (55.6)	0.871^†^
Dyslipidemia	25 (13.5)	10 (40.0)	15 (60.0)	0.725^†^
Anemia	18 (97.0)	9 (50.0)	9 (50.0)	0.543^†^
Diabetes	15 (8.1)	8 (53.3)	7 (46.7)	0.411^†^
Avascular necrosis	15 (8.1)	3 (20.0)	12 (80.0)	0.061^†^
Discoid	13 (7.0)	8 (61.5)	5 (38.5)	0.167^†^
Osteoporosis	12 (6.5)	7 (58.3)	5 (41.7)	0.275^†^
Premature menopause	12 (6.5)	4 (33.3)	8 (66.7)	0.474^†^
Rheumatoid arthritis	10 (5.4)	5 (50.0)	5 (50.0)	0.748‡
Chronic kidney disease	9 (4.9)	6 (66.7)	3 (33.3)	0.178‡
**Body Mass Index**				
Normal	91 (49.2)	29 (31.9)	62 (68.1)	0.008^†^
Underweight	31 (16.8)	16 (51.6)	15 (48.4)	
Overweight	63 (34.1)	35 (55.6)	28 (44.4)	
(Min = 14.52, Max = 60.40, Mean = 23.10, SD = 5.77)				

^†^the *p*-value associated with Chi-square test

^‡^*p*-value from Fisher’s Exact test

### Depressive symptoms and socio-demographic or clinical characteristics of the participants

**Fig. 2:**
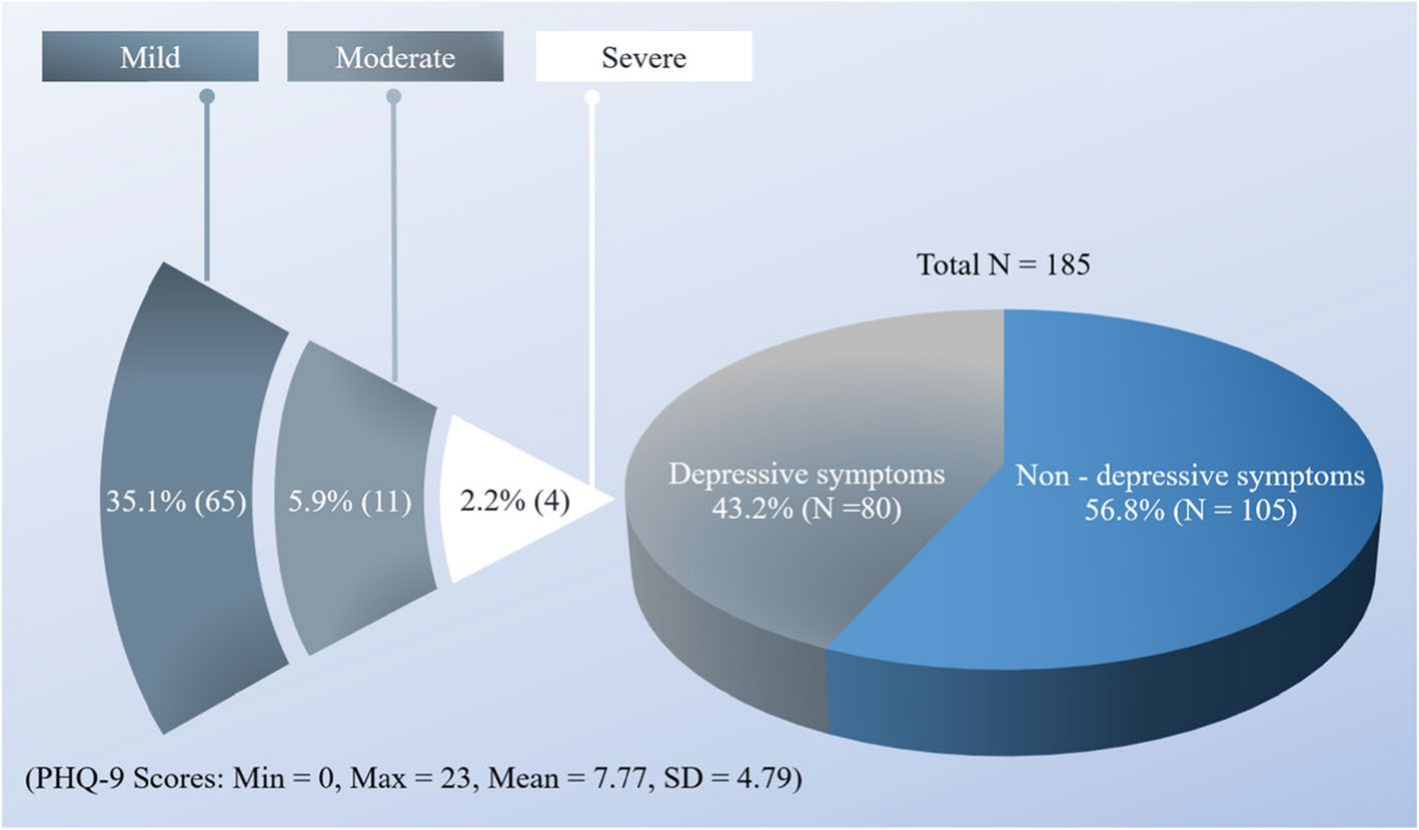
Prevalence of depressive symptoms in patients with SLE

The findings showed 43.2% of the participants had depressive symptoms. Classified by severity level of depressive symptoms, 2.2% of patients had severe depression, 5.9, and 35.1% had moderate or mild levels of depression. The PHQ-9 scores were between 0 and 23 points, with an average of 7.77 points (SD = 4.79) (Fig. 2).

Among the socio-demographic characteristics, sex, age, religion, education, occupation, right to access healthcare, family characteristics, family members, and monthly household income were not associated with depressive symptoms in patients with SLE. Household income sufficiency was significantly associated with depressive symptoms in patients with SLE (*p* = 0.008), and patients of different marital statuses tended to have different levels of depressive symptoms (*p* = 0.051) (Table 1).

Among the clinical characteristics, disease duration was not associated with depressive symptoms in patients with SLE. Prednisolone dosage (*p* = 0.004), disease flares especially both in the kidney system (*p* = 0.007) and muscles (*p* = 0.044), and body mass index (*p* = 0.008) were significantly associated with depressive symptoms in patients with SLE. Complications of avascular necrosis tended to be associated with depressive symptoms by patients (*p* = 0.061) (Table 2).

### Univariable logistic regression statistical analysis of depressive symptoms of the participants

Patients with disease flare (*p* = 0.002), organ damage (*p* = 0.036), moderate or severe pain (*p* = 0.044) (*p* = 0.002), fatigue (*p* = 0.000), taking prednisolone ≥15 mg daily (*p* = 0.004), moderate or low satisfaction with body image (*p* = 0.008) (*p* = 0.000), moderate or low social support (*p* = 0.001) (*p* = 0.002), and leftover of income (*p* = 0.009) was significantly associated with depressive symptoms in patients with SLE (Table 3).

**Table 3:** Univariable & Multivariable logistic regression analysis for depressive symptoms of the participants (*n* = 185)

Variables (N)	Univariable			Multivariable		
	cOR	95%CI	*p*-value	aOR	95%CI	*p*-value
**Biology factors:**						
**Disease flare (SLEDAI)**						
Non-disease flare (145)	1					
Disease flare (40)	3.13	1.51, 6.51	0.002**	1.59	.30, 8.32	0.585
(Min = 0, Max = 12, Mean = 1.96, SD = 3.03)						
**Organ damage (SLICC Damage Index)**						
Non-organ damage (95)	1					
Organ damage (90)	1.88	1.04, 3.38	0.036*	1.33	.60, 2.93	0.478
(Min = 0, Max = 5, Mean = 0.75, SD = 0.96)						
**Pain (NRS)**						
Non-pain (108)	1					
Mild pain (22)	.69	.25, 1.91	0.476	.61	.18, 2.07	0.425
Moderate pain (39)	2.15	1.02, 4.51	0.044*	1.53	.60, 3.91	0.379
Severe pain (16)	27.63	3.51, 217.32	0.002**	12.11	1.35, 108.46	0.026*
(Min = 0, Max = 9, Mean = 1.95, SD = 2.63)						
**Fatigue (FSS)**						
Non-fatigue (98)	1					
Fatigue (87)	4.53	2.43, 8.45	0.000***	2.36	1.08, 5.14	0.031*
(Min = 1, Max = 7, Mean = 3.63, SD = 1.54)						
**Prednisolone (mg/day)**						
0 (51)	1					
1–5 (89)	1.03	.50, 2.11	0.937	.98	.39, 2.48	0.979
6–14 (13)	2.93	.84, 10.30	0.093	3.68	.81, 16.67	0.091
≥ 15 (32)	4.03	1.57, 10.35	0.004**	5.75	1.76, 18.80	0.004**
(Min = 0, Max = 40, Mean = 5.90, SD = 7.71)						
**Psychology factor:**						
**Body image (BIS)**						
High satisfied (120)	1					
Moderate satisfied (46)	2.57	1.28, 5.15	0.008**	1.86	.77, 4.46	0.163
Low satisfied (19)	18.34	4.03, 83.43	0.000***	12.49	2.23, 69.80	0.004**
(Min = 0, Max = 20, Mean = 18.39, SD = 7.18)						
**Social factors:**						
**Social support (ESSI)**						
High social support (115)	1					
Moderate social support (57)	3.14	1.63, 6.08	0.001**	2.98	1.32, 6.74	0.013*
Low social support (13)	27.43	3.43, 219.18	0.002**	17.96	1.86, 173.77	0.009**
(Min = 9, Max = 30, Mean = 22.16, SD = 6.10)						
**Household income sufficiency**						
Leftover (90)	1					
No leftover (95)	2.22	1.23, 4.03	0.009**	1.63	.74, 3.56	0.225

*Abbreviations*: *SLEDAI* the Systemic Lupus Erythematosus Activity Index, *SLICC Damage Index* the Systemic Lupus International Collaborating Clinics Damage Index, *NRS* Numeric Rating Scale, *FSS* Fatigue Severity Scale, *BIS* Body Image Scale, *ESSI* the ENRICHD Social Support Instrument, *COR* Crude odds ratio, *AOR* Adjusted odds ratio, *95% CI* 95% confidence interval

^*^p-value < 0.05

^**^p-value < 0.01

^***^p-value < 0.001

### Multivariable logistic regression statistical analysis of depressive symptoms of the participants

Patients with severe pain (*p* = 0.026), fatigue (*p* = 0.031), taking prednisolone ≥15 mg daily (*p* = 0.004), low satisfaction with body image (*p* = 0.004), and moderate or low social support (*p* = 0.013) (*p* = 0.009) was significantly associated with depressive symptoms in patients with SLE. Patients who took prednisolone, 6 to 14 mg daily (*p* = 0.091), tended to show an associated with depressive symptoms (Table 3).

## Discussion

A decade ago, depressive symptoms were considered a significant mental health problem for the whole population but were under-recognized [57], especially for patients with chronic conditions. In the current study, the proportion of the participants with depressive symptoms was 43.2%, consistent with prior studies [2, 7, 9, 10, 23]. Compared with a previous study [58] conducted in a similar context during the COVID-19 outbreak in Southeast Asia, the proportion of moderate to severe depression in Thai patients with SLE was lower than those studied. This difference may have occurred because the measures of depressive symptoms in these studies differed. This study used PHQ-9, which is specific to screening for depressive disorders. In contrast, another study used the Depression Anxiety and Stress Scale (DASS-21), where researchers assessed depression by mental health status. Our patients rated 8.1 on severity of depressive symptoms, moderate to severe depressive symptoms diagnosed as depression higher than the general Thai population [59]. This severity rating gives us sufficient reason to be concerned about the depression in our patients with SLE.

According to the results of our study, biopsychosocial factors have more influence on depressive symptom severity in patients with SLE. Notwithstanding, the association between depressive symptoms and clinical symptoms has differed in past studies. Biology factors like disease flare, organ damage, pain, fatigue, and cumulative corticosteroid dosage were all involved [2, 27, 46]. Our study found a positive association between depressive symptoms and all those factors. However, the multivariable analysis findings did not show association with either disease flare or organ damage with depressive symptoms. Like previous studies, associations between depressive symptoms and disease flare are inconsistent because of methodological differences in measuring the disease activity of SLE. Using an objective disease activity measure such as SLEDIA, there was no association between depressive symptoms and disease flare [60–63]. Simultaneously, previous studies have suggested that patients and those with disease activity, including laboratory changes or disease damage, are more likely to have increased severity of depression in patients with SLE [7, 27].

Further research with larger sample sizes and well-controlled assessments of study methodology differences may clarify and confirm these phenomena. Besides risk factor of disease activity, depression in patients with SLE could be due to immune-mediated cognitive dysfunctions, which correlate with other pathological processes like autoantibodies (Abs), inflammatory markers, and micro vasculopathy, as well as volume reduction in the white matter and grey matter of the brain [64]. SLE’s specific cognitive impairments include attention, memory, and visuospatial process impairment and can cause mental disorders (delirium, dementia, mild cognitive impairment) [65]. Further studies defining the role of depression are essential to understand the pathophysiology of SLE-related cognitive dysfunction and depressive symptoms and to develop treatment strategies.

On the other hand, the observation in the multivariable analysis showed extreme pain, severe fatigue, and high dosage of steroid use were strongly interwoven in physical and psychiatric disorders among patients with SLE [27, 29, 38]. These factors and depressive symptoms share norepinephrine or serotonin neurotransmitter pathway pathology in the central nervous system that can provoke manifestations of physiological illness. Fatigue is associated with an increased risk for depression among these patients [29], and this study confirms these association findings. Heightened pain was associated with increased depressive symptoms in this study. This evidence also confirms various studies demonstrating that increased pain is related to a greater risk of depressive symptoms in patients with SLE [66, 67]. Depressive symptoms can also be caused by corticosteroid treatment through downregulation of brain-derived neurotrophic factors [68, 69]. In our study, patients who continued taking ≥15 mg prednisolone daily had significant depressive symptoms, comparable to 18.28 to 20 mg mean doses of prednisolone in a previous study in patients with major depressive disorder (MDD) [22, 27] and 15 mg daily for patients with non-major depressive disorder [22].

Increased body mass index scores in patients correlate with depressive symptoms, and the patients with depressive symptoms demonstrate higher levels of unemployment. Those findings could be confounding factors in depression but may also indicate the risk of depressive symptoms, which cause disease manifestations, disability, and productivity [29].

Dissatisfaction with one’s appearance is a real problem that patients with SLE frequently face [70, 71]. However, an understanding of SLE patients’ feelings about their body image has been lacking in Thai research. This omission makes it difficult to evaluate the degree of body image dissatisfaction, with much of the research on body image only emphasizing patients with breast cancer [72]. This gap needs to be addressed through focused research on these specific issues. From the previous studies, self-perceived appearance mediated the relationship between physical health-related quality of life and depression among patients with SLE [37, 73]. Our findings corroborated previous studies. In the present study, we found that low body-image satisfaction was an enormously significant predictor of depressive symptoms in patients with SLE, both in the univariable and multivariable analysis. Thus, a psychological intervention that targets belief and perception of body image in patients with SLE can reduce depressive symptoms.

Among social factors, the findings from the multivariable analysis did not show household income sufficiency was significantly associated with depressive symptoms in patients with SLE. A consistent previous examination found poverty was a significant predictor in the bivariate analysis, but not the multivariate [61]. However, social support is a crucial resource for patients with SLE with a high disease burden [74]. Many studies have noted the importance of social support regarding depression [75–77]. Good social support has been shown to protect from depression and elevate an individual’s emotional state [68]. It has also been established that those depressive characteristics are associated with decreased peer-related social support [75]. The univariable and multivariable analysis results found that social support was significantly associated with depressive symptoms in patients with SLE. These findings show that social support is vital for mental health and that a decrease in relationship satisfaction is an indicator of depressive symptoms.

Our findings shed light on the need for clinical implementation to reduce depression and improve the quality of life of SLE patients. A practical approach, including medication [78, 79] and psychosocial interventions, should provide biopsychosocial management [80, 81]. Further studies to figure out the effectiveness of the implementation for reducing depressive symptoms in patients with NPSLE, both pharmacological and non-pharmacological strategies, are necessary.

### Strengths and limitations

The strengths of this study include the use of psychological instruments, which are acceptable, reliable, and valid in the Thai populations. Moreover, independent variables were defined based on Engle’s biopsychological model. It is a practical way of understanding how patients suffer physical and mental illnesses from sociology to molecular biology [12, 82]. Regarding our result, preventive assessment of both psychosociological and somatic symptoms can be evaluated by clinical information and query but does not specify the neurological pathology of depression. Growing study for biomarker validation and pathophysiology elucidation for MDD gauging is incredibly challenging. Functional Near-Infrared spectroscopy (fNIRS) consistently demonstrated direct-attenuated cerebral hemodynamic changes in depressed with individual symptoms. Further evidence for fNIRS is shown in quantitative risk analysis and monitoring various treatment responses of patients with SLE who present with depressive symptoms [83, 84].

However, this present study has several limitations. Participants were selected using inclusion/exclusion criteria, and the investigation was performed at a single medical center. Therefore, it may not represent patients with initial treatment, psychiatric disorders, cognitive impairment, or life-threatening conditions. Additionally, the role of inflammation and genetic susceptibility for the emergence of depressive symptoms was not assessed. Thus, more psychoanalytic research is needed to clarify the relationship between the immune system of disease activity and a patient’s psychological function.

## Conclusion

In summary, depressive symptoms are highly prevalent among Thai patients with SLE. Depressive symptoms in patients arise from various causes, including particular perceptions of individual patients. Treatments of depressive symptoms may benefit patients with extreme pain, fatigue, high prednisolone dosage, low satisfaction of body image, and low social support. Further study of biopsychosocial factors is necessary to fully address the causes and potential management of debilitating depression in patients with SLE.

## Data Availability

The datasets generated and/or analysed during the current study are not publicly available due to the used data protection declaration, but are available from the corresponding author on reasonable request.

## Abbreviations

SLE: Systemic Lupus Erythematosus
PHQ-9: The Patient Health Questionnaire-9
BPS: The Biopsychosocial Model
SLEDAI: The Systemic Lupus Erythematosus Activity Index
SLICC Damage Index: The Systemic Lupus International Collaborating Clinics Damage Index
NRS: Numeric Rating Scale
FSS: Fatigue Severity Scale
BIS: Body Image Scale
ESSI: The ENRICHD Social Support Instrument
NCDs: Non-communicable Diseases
CNS: Central Nervous System
NPSLE: Neuropsychiatric Systemic Lupus Erythematosus
ACR: American College of Rheumatology
NSAIDs: Non-Steroidal Anti-Inflammatory Drugs
NSO: The National Statistical Office of Thailand
ICD-10: International Classification of Diseases 10th Revision
COVID-19: Coronavirus Disease of 2019
SPSS: Statistical Package for the Social Sciences
MDD: Major Depressive Disorder
fNIRS: Functional Near-Infrared spectroscopy
